# Disinfection Efficiency Among Black Alder and Hybrid Alder Genotypes and Their Influence on Alder Seed Germination In Vitro

**DOI:** 10.1111/1758-2229.70183

**Published:** 2025-09-19

**Authors:** Vytautas Čėsna, Ieva Čėsnienė, Virgilijus Baliuckas, Jonas Žiauka

**Affiliations:** ^1^ Institute of Forestry Lithuanian Research Centre for Agriculture and Forestry Kaunas Lithuania; ^2^ Faculty of Natural Sciences Vytautas Magnus University Kaunas Lithuania

**Keywords:** *Alnus*, *Cladosporium*, deciduous, in vitro, micropropagation, seeds

## Abstract

The presented study aimed to test a set of *Alnus* genotypes, including hybrids of black and grey alders ( *A. glutinosa* × *A. incana* ) and *A. glutinosa,* based on their spring phenology, using in vitro screening assays. We evaluated the disinfection efficiency and the susceptibility of the surviving fungi in different *Alnus* genotypes, comprising five with early spring phenology, four with late spring phenology and four hybrids. Then, the best‐performing explants of four *Alnus* genotypes, including at least one of early and late spring phenology, as well as one hybrid, were selected to evaluate the explant influence on other *Alnus* seed germination under stress from the fungus *Cladosporium cladosporioides* inoculum. The explants of *Alnus* hybrids 047 and 026 were characterised by stronger leaf development than the *A. glutinosa* genotype 19–43–8 K, which, however, displayed the highest success of explant sterilisation. The explants of *A. glutinosa* genotype 19–43–8 K and of *Alnus* hybrid 026 were shown to increase germination of neighbouring (planted in the same test tube) *Alnus* seeds and seedling development, especially root system expansion under the stress from *C. cladosporioides*. These results point to the potential of the selected *Alnus* genotypes to relieve biotic stress in other *Alnus* seeds.

## Graphical abstract

Natural hybrids of black and grey alders can be characterised by higher resistance to biotic and abiotic stressors. The explants of alder hybrid 026 were characterised by stronger leaf development and were shown to increase germination of neighbouring alder seeds and seedling development under the stress from fungi *Cladosporium cladosporioides*.

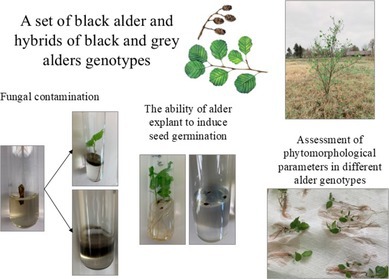

## 1 Introduction

Black alder ( *Alnus glutinosa* (L.) Gaertn.) and grey alder ( *Alnus incana* (L.) Moench) are pioneer tree species in riparian habitats, with significant ecological importance and a natural distribution across all of Europe (Claessens et al. 2010; Martín et al. 2024). Due to climate warming, the distribution ranges of different tree species can change, leading to more frequent co‐occurrence and hybridisation, which is relevant for biodiversity (Li et al. 2021). Abiotic stresses, such as desiccation and moisture, may lead to the formation of integrated gene pools of *A. glutinosa* × *A. incana* , which may be adaptive for better resistance against these environmental factors (Jurkšienė et al. 2021). Introgressive hybridisation can benefit forest evolution by creating new genotypes (Jurkšienė et al. 2021; Neale and Wheeler 2019). Increased tree genetic diversity enables them to adapt more quickly to constantly changing environmental conditions that pose numerous threats, such as habitat loss, pollution or fragmentation (Aravanopoulos 2018; Fady et al. 2022; Martín et al. 2024). Due to the heterosis effect, natural hybrids of *A. glutinosa* × *A. incana* can be characterised by better wood mechanical properties, higher resistance to biotic and abiotic stressors, including *Phytopthora* root rot and drought, and synthesis of higher amounts of proteins (Marković et al. 2024; Vanden Heuvel 2011).

Nevertheless, tree hybridisation can also negatively influence alders by causing genetic erosion, potentially leading to their extinction (Jurkšienė et al. 2021; Soltis and Soltis 2009). Moreover, *Alnus* hybridisation is further challenged by additional threats, including its tolerance to locally present pathogenic fungi, which may facilitate the spread of fungal infections within hybrid populations. For instance, the emergence of the hybrid oomycete species complex *Phytophthora* × *alni*, along with the related non‐hybrid species *P. plurivora* and *P. lacustris* , has been linked to significant declines in *Alnus* populations across Europe (Aguayo et al. 2014; Brasier et al. 2004; Bregant et al. 2020, 2024; Jung et al. 2018; Martín et al. 2024). Other pathogens that cause severe damage to deciduous trees, especially during their young age, are fungal species of the genus *Cladosporium*. These fungi affect tree roots and lead to poor nitrogen exchanges between trees and the rhizosphere (Kranjec Orlović et al. 2020; Põlme et al. 2013). *Cladosporium cladosporioides* is a common fungal species with a broad ecological range, including functioning as an endophyte in various plant hosts. Previous studies have identified it as a natural endophyte in *Alnus* species, including older or mature alder trees, where it is typically asymptomatic and not considered harmful (Kowalski and Kehr 1992). However, under certain conditions, particularly in young, stressed, or immunologically immature trees such as seedlings or saplings in seed orchards, *C. cladosporioides* may act opportunistically and exhibit mild pathogenicity (Dutta et al. 2014). As soil and climate conditions shift in ways that increasingly favour pathogen attacks on alders (Redondo et al. 2018), there is a growing need to study the genetic diversity of tree hybrids to assess their response to these threats (Martín et al. 2024).

Trees shape ecosystem dynamics via allelopathy, a biochemical process in which plants release secondary metabolites to influence the growth and development of neighbouring organisms (Fernandez et al. 2016). These effects are primarily attributed to phenolics and flavonoids, which can be released into the environment through leaf litter, root exudates and decomposing plant material (John and Sarada 2012). Furthermore, certain species of plants, including trees, can develop complex systems that let them interact with nitrogen‐fixing bacteria (Ballhorn et al. 2017). Alders form a root‐based symbiosis with nitrogen‐fixing actinobacteria of the genus *Frankia* (Ballhorn et al. 2017; Hay et al. 2020). The actinobacteria can reduce dinitrogen into ammonium, which can be directly exported through the ammonium transporter AmtB of tree cells (Prell and Poole 2006). For nitrogen storage, trees can develop specialised organs called nodules, in which symbiotic interactions between host plants and *Frankia* spp. have recently been investigated (Gifford et al. 2019; Pesce et al. 2019). The plant–bacteria symbiosis also benefits the forest ecosystem, enriching the rhizosphere (Bühlmann et al. 2017; Sen et al. 2022). In exchange for the ammonium, the actinobacteria receive carbon, energy and a protected niche from a host plant (Mathesius 2022; Puri 2020). Understanding the symbiotic mechanisms and how alders can influence seed germination is essential for forest management, conservation, and ecological restoration.

Using in vitro screening assays, we can perform studies on disinfection efficiency, plant growth and the ability to induce the development of other plants (da Silva et al. 2015). We hypothesised that different genotypes and spring phenology of *A. glutinosa* and alder hybrids vary in their susceptibility to fungal contamination and their capacity to influence seed germination and seedling development. To test this hypothesis, the present study focused on two main objectives: (Aguayo et al. 2014) to assess variation in fungal contamination levels and leaf emergence among *A. glutinosa* and hybrid alder genotypes following a standardised surface disinfection protocol, to improve micropropagation success; (Altschul et al. 1990) to evaluate how explants from different genotypes affect seed germination rates and seedling morphological parameters under in vitro conditions.

## 2 Materials and Methods

### 2.1 Plant Material and Objects

Seeds and vegetative buds from 13 different genotypes of *Alnus* were collected from 5‐year‐old trees growing in a seed orchard located in Josvainiai Eldership, Kėdainiai District, central Lithuania. A total of 28 *A. glutinosa* clones, 12 hybrid clones, and 19 *A. incana* clones were planted in the forestry plantation. Between two and nine trees of each clone were planted. The planting spacing was 5 × 8 m. For experimental analysis, only those *Alnus* genotypes were selected that exhibited extreme values in spring phenology points (early or late spring phenology), as determined by researchers from the Forest Genetics and Tree Breeding Department of the LAMMC in 2017. The spring phenology was assessed in the seed orchard based on alder leaf flushing on a scale, ranging from 1 (low: bud just beginning to burst) to 5 (high: leaves are green and hold firmly). The sampling was performed in November, when environmental conditions were characterised by cool temperatures (4°C–5°C) and moderate humidity, with monthly precipitation averaging 20–40 mm. All selected trees appeared healthy and free of visible disease symptoms. Five different genotypes of *A. glutinosa* with high points of spring phenology (H), four *A. glutinosa* genotypes with low points of spring phenology (L), and four hybrids of *A. glutinosa* × *A. incana* (Hb) were selected (Table 1).

**TABLE 1:** Alder (*Alnus*) genotypes based on their spring phenology.

*A. glutinosa* /spring phenology points		*A*. × *glutinosa* × *A. incana* /spring phenology points
13–99–1 K (H1)/4.8	4–139–4 K (L1)/2.2	041 (Hb1)/3.8
41–91–7 K (H2)/4.5	26–133–9 K (L2)/2.5	047 (Hb2)/4.3
23–77–5 K (H3)/4.3	4–98–8 K (L3)/2.5	026 (Hb3)/2.8
23–95–5 K (H4)/4.3	19–43–8 K (L4)/2.8	048 (Hb4)/3.0
4–139–7 K (H5)/4.0		

Abbreviations: H = *A. glutinosa* with high spring phenology (early spring phenology); Hb = hybrids of *A. glutinosa* × *A. incana* ; L = *A. glutinosa* with low spring phenology (late spring phenology).

### 2.2 Experimental Design

#### 2.2.1 Disinfection and Planting of Alnus Vegetative Explants

**FIGURE 1:**
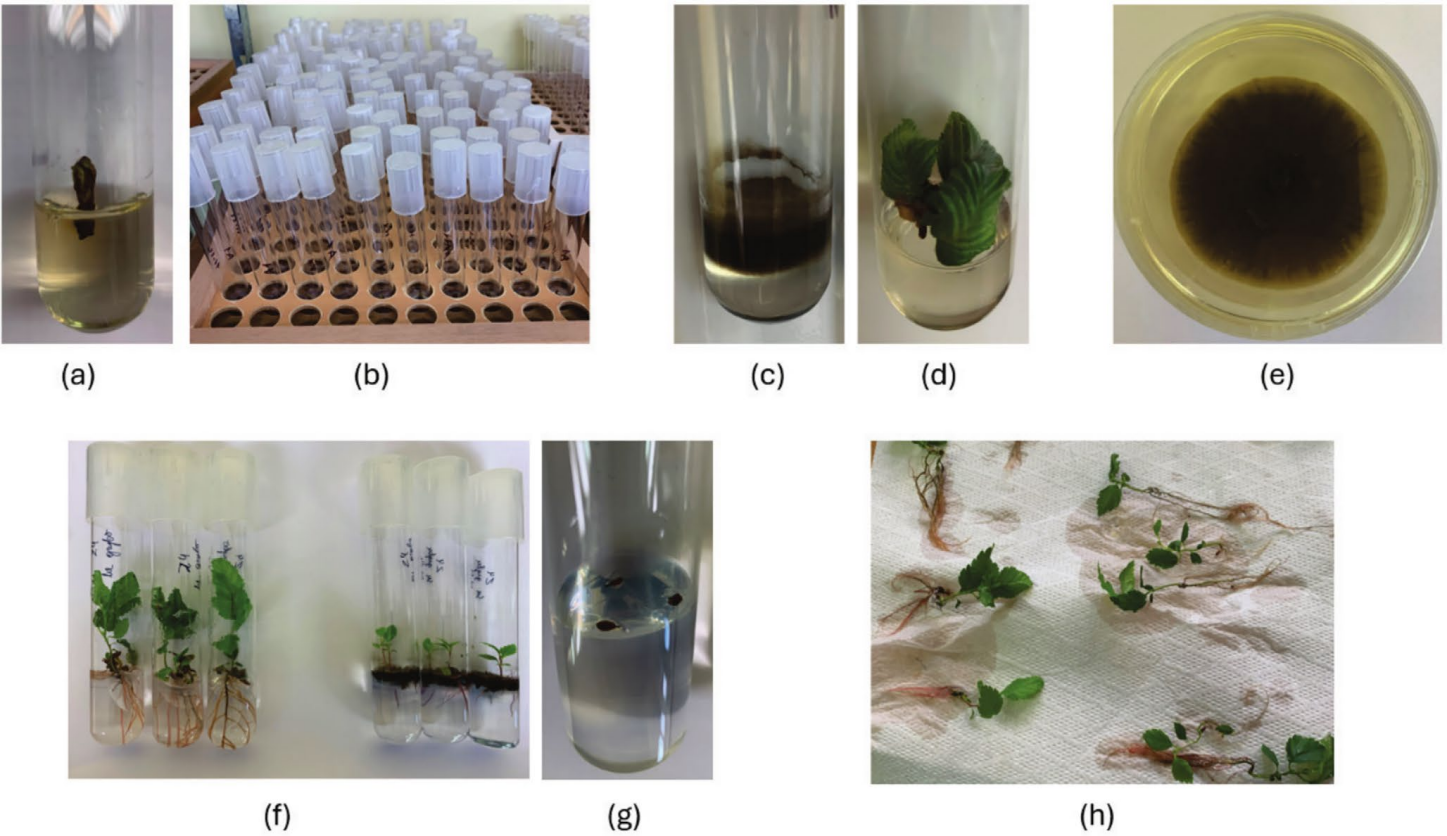
The experimental design: (a) and (b) disinfected *Alnus* explants planted in cylindrical closed tubes into autoclaved WPM, using in vitro screening assays; (c) fungal contamination in the WPM within *Alnus* explant; (d) *Alnus* explant without the contamination; (e) *Cladosporium cladosporioides* multiplication by growing in Petri dishes; (f) three *Alnus s*eeds planted together with *Alnus* explant (on the left—without *C. cladosporium* inoculum; on the right—with *C. cladosporium* inoculum); (g) planting of *Alnus* seeds without *Alnus* explant (control); (h) *Alnus* plants being removed from the cylindrical tubes and the phytomorphological parameters being measured.

Forty *Alnus* vegetative buds from 13 different genotypes (*n* = 520) were stored in a fridge (+4°C) for 7 days after the collection. Then the explants were disinfected by chemical solvents: Ace (50% commercial bleach) (Dalli Production, Romania SRL) for 2 min, 75% ethanol (Stumbras, Lithuania) for 3 min, and 0.1% AgNO_3_ (Haarlem, the Netherlands) for 3 min, using sterile syringes. After each chemical, the explants were soaked in disinfected distilled H_2_O (for 2 min after Ace, 3 × 3 min after ethanol, and 3 min after AgNO_3_). After disinfection, the explants were planted in cylindrical tubes, closed with colourless plastic caps, into 5 mL of autoclaved Woody Plant Medium (WPM) for in vitro screening (Figure 1a,b). Glass culture tubes for growing *Alnus* explants were 20 mm in diameter and 150 mm in height with 5 mL WPM.

WPM (Lloyd and McCown 1980) was used as a nutrient medium and given by a basal salt mixture (Sigma‐Aldrich Chemie GmbH, Germany) with standard concentrations of vitamins (Duchefa Biochemie, the Netherlands), 20 g × L^−1^ sucrose (Duchefa Biochemie, the Netherlands), and 4 g × L^−1^ gelrite (Duchefa Biochemie, the Netherlands). Pilot studies using WPM pH values of 3.7, 4.3, 4.9, and 5.5 were conducted (Table S1). The best pH value for seed germination and lowest contamination was 4.9. Thus, this pH value was used in this study for further experiments.

#### 2.2.2 Fungal Contamination and Alnus Phytomorphological Measurements

The frequency of fungal contamination for vegetative *Alnus* explants in each of the 13 *Alnus* genotypes was estimated at 1, 2 and 4 weeks after the explants had been planted into WPM (Figure 1c,d). The frequency of leaf emergence of *Alnus* explants was estimated at 2 and 4 weeks after the explants had been planted into WPM. The number of leaves per explant and the largest leaf width were estimated 8 weeks after planting.

#### 2.2.3 Fungal Multiplication and Identification

The fungal species most frequently associated with contamination of *Alnus* explants was selected for further analysis. Cultures were transferred from cylindrical culture tubes to sterile Petri dishes containing potato dextrose agar (PDA) by aseptically excising a mycelial plug and placing it at the centre of the agar surface under laminar airflow conditions.

To identify the fungal species isolated from *Alnus* explants, DNA was extracted from pure fungal cultures using a CTAB protocol optimised for fungal tissue (Rosling et al. 2003). DNA concentration and purity were assessed using a NanoDrop One spectrophotometer (Thermo Scientific, Rochester, NY, USA), and samples with a 260/280 ratio between 1.8 and 2.0 were used for downstream analyses. The internal transcribed spacer (ITS) region of the ribosomal DNA, a widely used fungal barcode, was amplified using the universal primers ITS1 and ITS4 (Martin and Rygiewicz 2005). The PCR was initiated with an initial denaturation step at 95°C for 5 min, followed by 30 cycles of denaturation at 95°C for 30 s, annealing at 56°C for 30 s and extension at 72°C for 30 s, culminating in a final extension step at 72°C for 7 min. The products were purified and sent for bidirectional Sanger sequencing at the Macrogen sequencing centre (Amsterdam, Netherlands). Sequence chromatograms were analysed using Chromas 2.6.6, and reverse reads were transformed to complementary sequences for alignment with forward reads. Manual sequence correction and consensus building were performed using BioEdit 7.2.5. Taxonomic identification was conducted by comparing the resulting consensus sequences against the NCBI GenBank database using the BLASTn algorithm (Altschul et al. 1990). The fungal isolate was identified as *C. cladosporioides*, with 100% sequence identity and 100% query coverage to reference sequences. A representative ITS sequence has been deposited in GenBank under accession number MT609901.1.

#### 2.2.4 Alnus Explant Influence on Seed Germination

Three different genotypes of *A. glutinosa* 23–95–5 K (H4), 4–139–4 K (L1), 19–43–8 K (L4) and one of *A. glutinosa* × *A. incana* hybrid 026 (Hb3) were selected for further assessment of the explant's germination enhancement and induced defence to pathogenic fungus *C. cladosporioides* of other *Alnus* seeds. The genotypes were chosen based on explant susceptibility to contamination, selecting at least one genotype from each spring phenology group and hybrid alder, representing those with the best performance. The explants were transferred to new, same‐type cylindrical tubes with fresh autoclaved WPM. The explants were screened for *C. cladosporioides* prior to inoculation, and only those free of the fungus were used. *Alnus* seeds were collected from the same seed orchard and at the same time as the buds. They were stored in a fridge (+4°C) for 8 weeks after collection before being planted into WPM medium. The *Alnus* seeds were disinfected with 96% ethanol (Stumbras, Lithuania) for 3 min. Using a glass stick, one drop of detergent Tween (Duchefa Biochemie, the Netherlands) was added, and the seeds were soaked in 30% H_2_O_2_ (Sigma‐Aldrich Chemie GmbH, Germany) for 2 × 80 min. Then, three *Alnus* seeds were planted into WPM in each tube, being positioned at 60° angles around one 7‐week‐old growing *Alnus* explant in the middle. Half of the cylindrical tubes with each genotype *Alnus* explant were inoculated with the fungus *C. cladosporioides* (3 seeds × 13 tubes [eight with *C. cladosporioides* inoculum and eight without the fungal inoculum] × 5 variables [Control; H4, L1, L4, Hb3]; *n* = 240) (Figure 1f). For the control, three *Alnus* seeds were planted into WPM without *Alnus* explant in the middle (Figure 1g). Seed germination was assessed 1 and 2 weeks after planting. The shoot length, primary root length and the longest lateral root length were measured with a ruler, and the number of lateral roots was assessed 8 weeks after planting (Figure 1h).

### 2.3 Statistical Analysis

R (version 4.2.1) with RStudio (version 1.1.456) and Microsoft Excel for Microsoft 365 was used for the data analysis.

The fungal contamination status of each vegetative *Alnus* explant (contaminated = 1, uncontaminated = 0) was recorded for 13 genotypes across three timepoints: 1, 2 and 4 weeks after planting into WPM medium. Genotypes were classified into three broad groups (H, L and Hb) based on species and spring phenology. The frequency of fungal contamination was calculated for each genotype and each broad group at each time point. For group‐wise comparisons, pairwise Fisher's exact tests (Eisinga et al. 2017) were conducted among genotype broad groups and individual genotypes. *p* Values were adjusted for multiple comparisons using the Benjamini–Hochberg (BH) method (Bogdan et al. 2008). Significance groupings were assigned using the *multcompView* package (Graves et al. 2015), and letters indicating statistically similar or different groups (*α* = 0.05) were overlaid on the bar plots. Bar plots were generated using *ggplot2* (Wickham et al. 2016), displaying mean contamination rates with standard error bars.

Leaf emergence frequency data were treated as binary outcomes (1 = presence; 0 = absence). For each genotype and time point (2 weeks and 4 weeks after planting), the proportion of explants with leaf emergence was calculated and compared between contaminated and non‐contaminated explants. The frequency of leaf emergence was calculated for each genotype and broad group at each time point using the same statistical method as that for calculating the frequency of fungal contamination.

To assess differences among genotypes on the number of *Alnus* leaves and the largest leaf width, a Kruskal–Wallis test (McKight and Najab 2010) was applied separately for both variables. Dunn's test with Bonferroni correction (Dinno and Dinno 2017) was performed as a post hoc test to determine pairwise differences. Results were summarised using compact letter displays, where different letters indicate statistically significant differences (*p* < 0.05) among genotype × contamination groups (contaminated or not‐contaminated). The effect of broader genetic groups (H, L, Hb) on both traits was assessed using Kruskal–Wallis tests.

To assess the effects of different *Alnus* genotypes on seed germination and plant morphological traits in vitro, four genotypes (H4, L1, L4 and Hb3) were selected. Half of the explants were inoculated with the fungi *C. cladosporioides*. Statistical significance (*p* < 0.05) was determined using Fisher's exact test with BH correction and results were visualised with standard error bars and letter‐based groupings.

The effects of genotype, *C. cladosporioides* inoculation, and their interaction on plant phytomorphological parameters were evaluated using a two‐way analysis of variance (ANOVA) (Kim 2017). When the ANOVA indicated significant effects (*p* < 0.05), Tukey's honest significant difference (HSD) post hoc test was applied to determine pairwise differences. Mean values were reported with their corresponding SE, and statistically distinct groups were annotated using lowercase letter displays (*α* = 0.05).

Morphological parameters of *Alnus* plants, including shoot length, primary root length, number of lateral roots and lateral root length, were used for a principal component analysis (PCA). PCA was performed using the *prcomp* (Yuan et al. 2016) function, with variables scaled and centred to ensure equal contribution regardless of units or magnitude. PCA was used to reduce dimensionality and assess patterns of variation among samples associated with fungal inoculation (*C. cladosporioides*) versus non‐inoculated controls. The PCA biplot was generated using the *autoplot* function from the *ggfortify* (Yuan et al. 2016) package. Samples were represented as X‐shaped points, colour‐coded based on inoculation status. Trait contributions were visualised through black loading vectors and labels, indicating the direction and strength of each parameter's influence on the principal components. All plotting was done using the *ggplot2* package.

## 3 Results

### 3.1 The Frequency of Fungal Contamination

**FIGURE 2:**
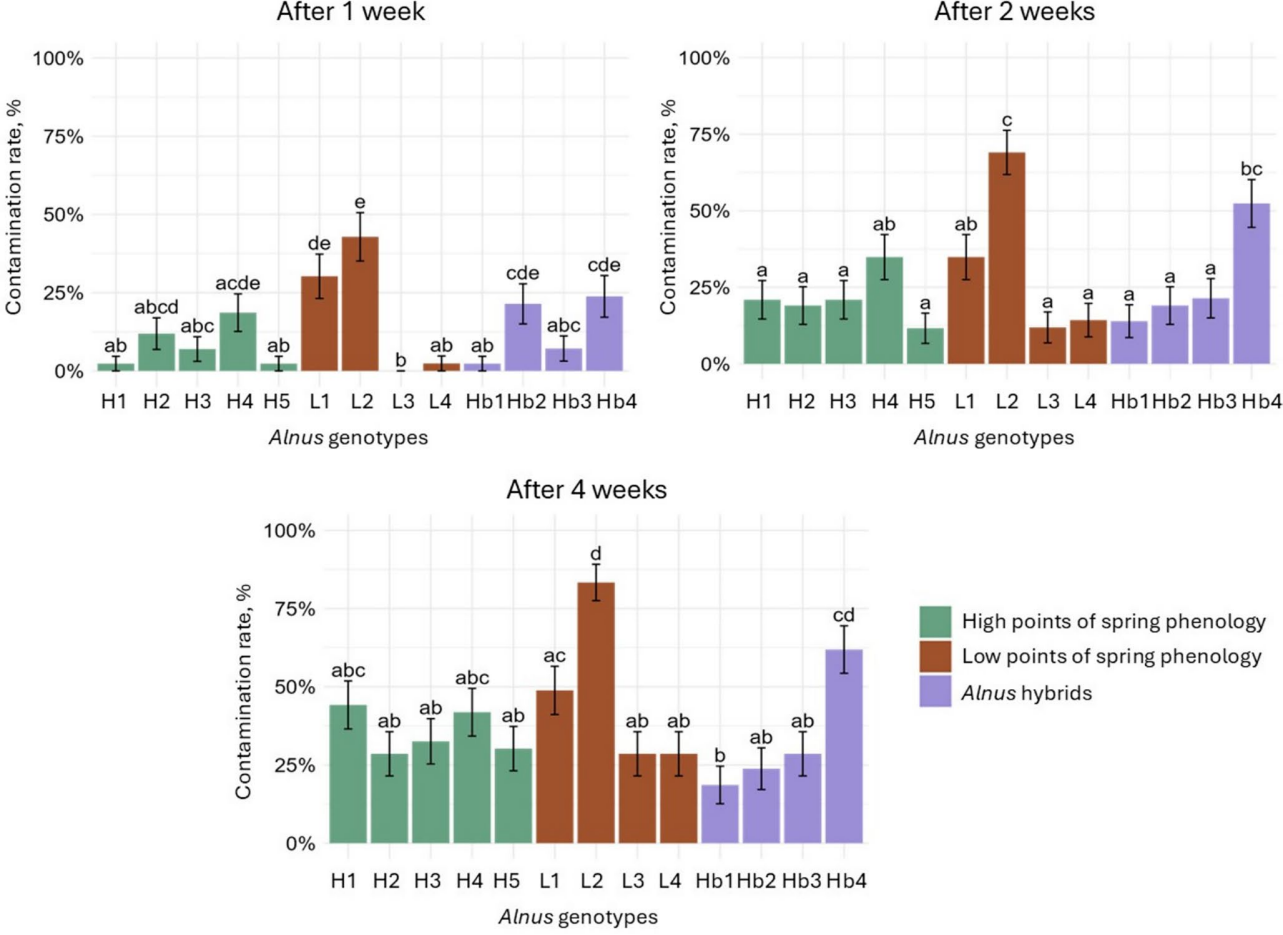
Frequency of fungal contamination (%) in different genotypes of vegetative *Alnus* explants at 1, 2 and 4 weeks after planting into WPM medium. Genotype codes (H, L, Hb) are described in Table 1. H = *Alnus* *glutinosa* with high points of spring phenology (early spring phenology); Hb = hybrids of *A. glutinosa × A. incana*; L = *A. glutinosa* with low points of spring phenology (late phenology). Statistically significant differences (*p* < 0.05) are shown with different letters above error bars representing standard errors (SE). For statistical analysis, Fisher's exact test and the Benjamini–Hochberg (BH) multiple comparisons adjustment were used.

The frequency of the pathogenic fungi contamination on vegetative *Alnus* explants in each of the 13 genotypes was assessed 1, 2 and 4 weeks after the explants had been planted in vitro into WPM. No significant differences in fungal contamination rates were observed between the broad genotype groups (H, L, and Hb) at 1, 2 or 4 weeks after planting (*p* > 0.05) (Figure S1). The most extensive fungal contamination was determined in three different *Alnus* genotypes: 26–133–9 K (L2), 4–139–4 K (L1) and *Alnus* hybrid 048 (Hb4) throughout all observations: after 1 week—L2 42.9%, L1 30.2% and Hb4 23.8%; after 2 weeks—L2 69.1%, L1 34.9% and Hb4 52.4%; after 4 weeks—L2 83.3%, L1 48.8% and Hb4 61.9% (Figure 2). On the contrary, the least fungal contamination 4 weeks after planting was determined in *Alnus* hybrid genotypes 041 (Hb1) and 047 (Hb2) (the frequency of fungal contamination was 18.6% and 23.8%, respectively).

### 3.2 Alnus Leaf Development

**FIGURE 3:**
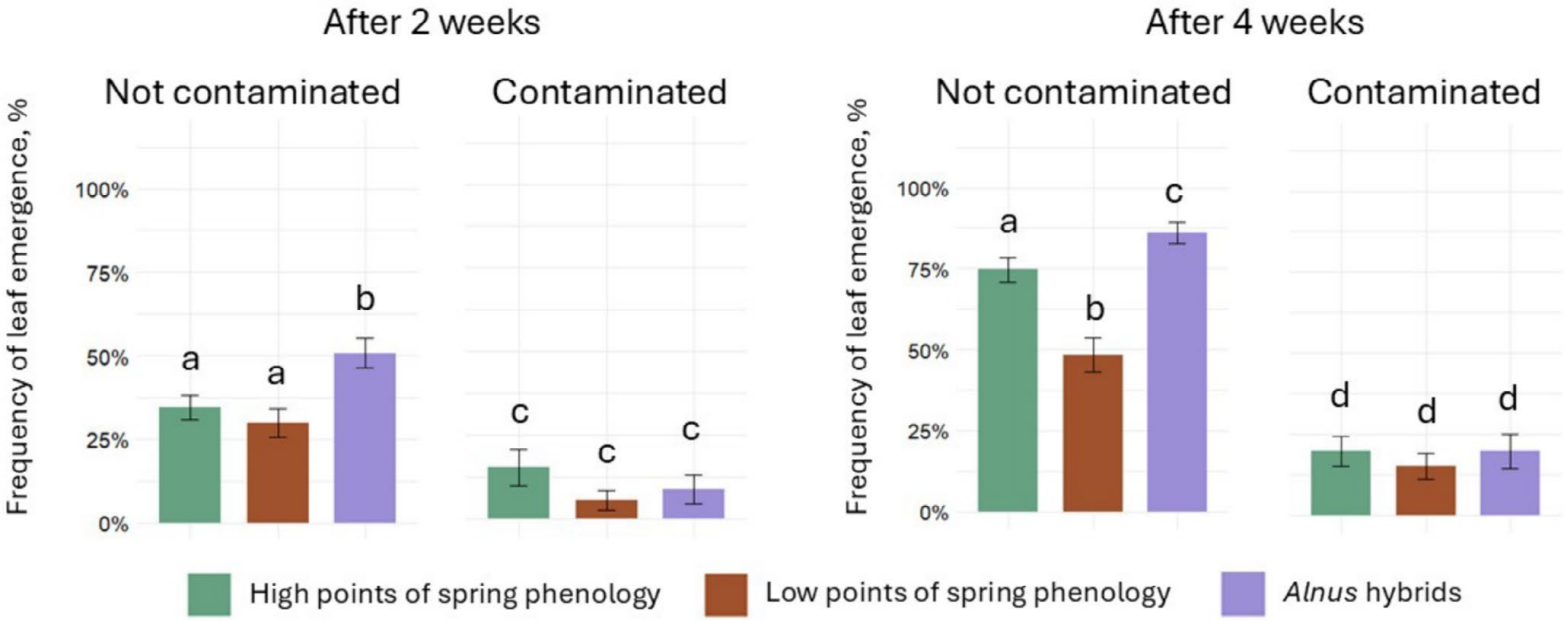
Frequency of leaf emergence (%) in different broad genotype groups (different colours) of vegetative *Alnus* explants: (Aguayo et al. 2014) *A. glutinosa* with high points of spring phenology (early spring phenology); (Altschul et al. 1990) *A. glutinosa* with low points of spring phenology (late phenology); (Aravanopoulos 2018) hybrids of *A. glutinosa × A. incana* at 2 and 4 weeks after planting into WPM medium. Statistically significant differences (*p* < 0.05) are shown with different letters above error bars representing standard errors (SE). For statistical analysis, Fisher's exact test and the Benjamini–Hochberg (BH) multiple comparisons adjustment were used.

To evaluate the effects of explant spring phenology, genotype and fungal contamination on leaf development in *Alnus* explants, the frequency of leaf emergence was assessed 2 and 4 weeks after transfer to WPM medium. At both time points, fungal contamination significantly (*p* < 0.05) reduced leaf emergence across all broad genotype groups (Figure 3). After 2 weeks, non‐contaminated *Alnus* hybrids showed the highest frequency of leaf emergence (50.8% ± 4.5%), significantly outperforming both early spring phenology (34.5% ± 3.7%) and late spring phenology *A. glutinosa* genotypes (29.8% ± 4.3%). Among contaminated explants, emergence rates dropped markedly, with all groups ranging between 5.5% and 15.2%, showing no statistically significant differences. At 4 weeks, the pattern was more pronounced (Figure 3). Non‐contaminated *Alnus* hybrids reached a leaf emergence frequency of 85.8% ± 3.3%, significantly (*p* < 0.05) higher than early phenology *A. glutinosa* (74.6% ± 3.7%) and late phenology genotypes (48.3% ± 5.3%). In contrast, contaminated explants again showed uniformly low leaf emergence frequencies, with no significant differences among groups, ranging from 15.0% to 19.7%.

**FIGURE 4:**
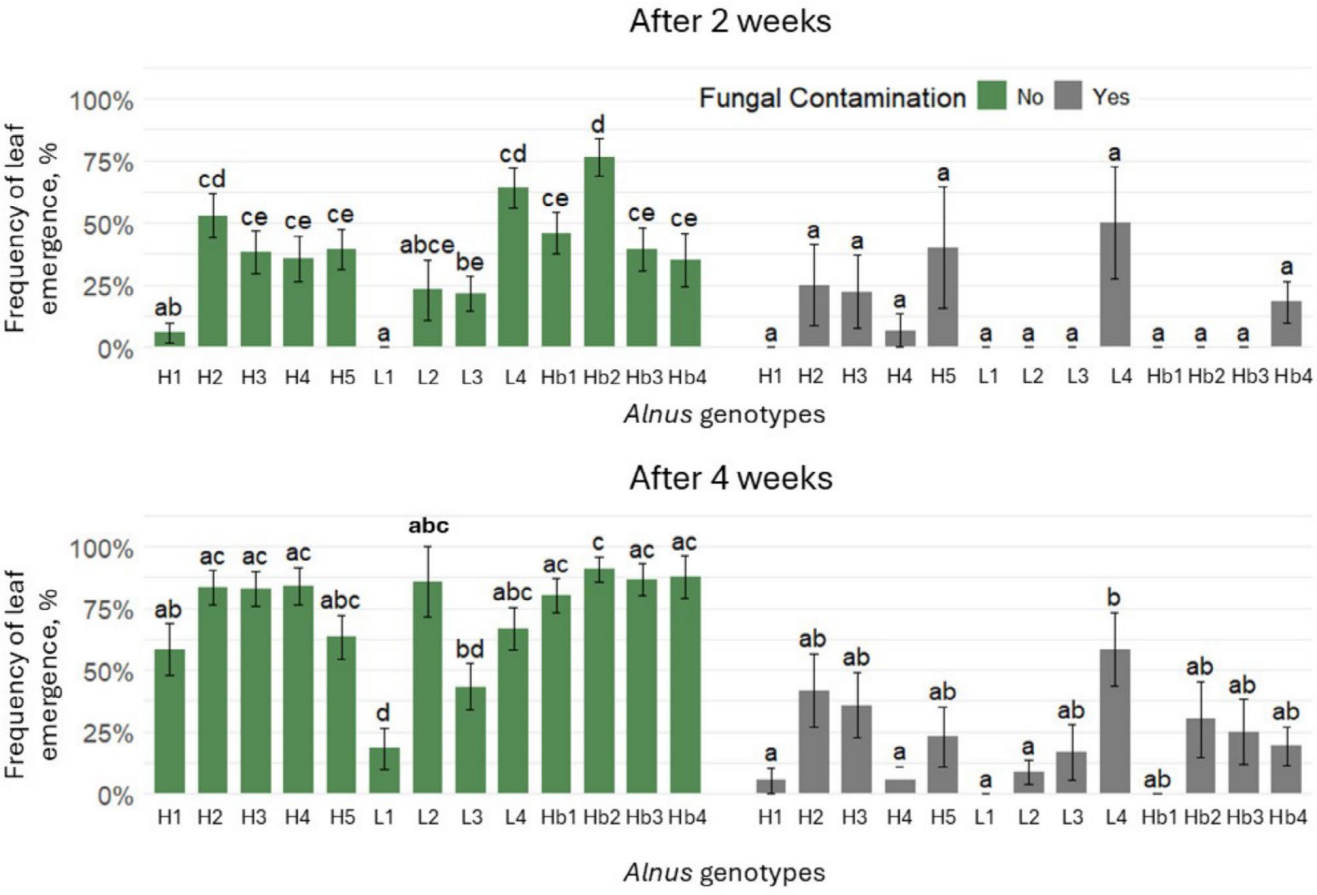
Frequency of leaf emergence (%) in different genotypes of vegetative *Alnus* explants (not‐contaminated and contaminated) at 1, 2 and 4 weeks after planting into WPM medium. Genotype codes (H, L, Hb) are described in Table 1. H = *Alnus glutinosa* with high points of spring phenology (early spring phenology); Hb = hybrids of *A. glutinosa ×* *A.* incana; L = *A. glutinosa* with low points of spring phenology (late phenology). Statistically significant differences (*p* < 0.05) are shown with different letters above error bars representing standard errors (SE). For statistical analysis, Fisher's exact test and the Benjamini–Hochberg (BH) multiple comparisons adjustment were used.

Analysing leaf development among different *Alnus* genotypes revealed that fungal contamination had a significant impact (*p* < 0.05) on leaf emergence 2 weeks after planting (Figure 4). Specifically, the frequency of leaf emergence was significantly reduced in genotypes 23–95–5 K (H4) (by 30.8%), 4–98–8 K (L3) (by 22.2%), 041 (Hb1) (by 47.2%), 047 (Hb2) (by 64.6%) and 026 (Hb3) (by 39.4%). At 4 weeks after planting, pathogenic fungi continued to negatively affect leaf emergence in nearly all genotypes, except for 19–43–8 K (L4), which showed no meaningful difference between contaminated (69.2%) and uncontaminated (69.0%) explants (Figure 4). The most pronounced reductions in leaf emergence at this stage were observed in genotypes 23–95–5 K (H4) and 048 (Hb4), with decreases of 78.4% and 68.3%, respectively.

The number of leaves per *Alnus* explant and the largest leaf width were evaluated 8 weeks after the explants had been planted into WPM. No significant differences in the number of leaves per explant and the largest leaf width were observed between the broad genotype groups (H, L and Hb) at either non‐contaminated or contaminated samples (*p* > 0.05) (Figure S2). The explants of the genotype 19–43–8 K (L4) of *A. glutinosa* were characterised by the highest number of leaves per explant in both experimental variables—non‐contaminated and contaminated (4.7 ± 0.4 leaves per explant in non‐contaminated and 3.3 ± 0.2 leaves per explant in contaminated) (Table 2). The explants of the genotype 4–139–4 K (L1) of *A. glutinosa* had only 2.2 ± 0.2 leaves per explant in non‐contaminated and 1.0 ± 0.2 leaves per explant in contaminated samples. The *A. glutinosa* explants of the L4 genotype had the highest leaf width, with an average of 7.3 ± 0.5 mm in non‐contaminated and 7.1 ± 0.7 mm in contaminated.

**TABLE 2:** The number of leaves per *Alnus* explant and the largest leaf width in different genotypes of vegetative *Alnus* explants (not‐contaminated and contaminated) at 8 weeks after planting into WPM medium.

*Alnus* genotype	Number of leaves per explant		The largest leaf width, mm	
	Non‐contaminated	Contaminated	Non‐contaminated	Contaminated
H1	2.7 ± 0.4^ab^	1.7 ± 0.2^ab^	7.1 ± 0.9^abc^	5.1 ± 0.7^abc^
H2	3.7 ± 0.3^bc^	2.0 ± 0.3^abc^	6.4 ± 0.4^abc^	5.6 ± 0.7^abc^
H3	3.5 ± 0.4^ab^	1.9 ± 0.2^abc^	6.7 ± 0.7^ac^	5.8 ± 0.7^b^
H4	3.9 ± 0.6^abc^	1.7 ± 0.3^abc^	5.6 ± 0.5^abc^	4.5 ± 0.5^abc^
H5	2.5 ± 0.2^ab^	1.9 ± 0.2^ab^	5.4 ± 0.4^abc^	5.2 ± 0.5^abc^
L1	2.2 ± 0.2^ab^	1.0 ± 0.2^a^	4.8 ± 0.9^abc^	4.1 ± 0.6^ac^
L2	4.0 ± 0.6^abc^	1.3 ± 0.3^ab^	5.7 ± 0.7^abc^	6.3 ± 0.8^abc^
L3	3.3 ± 0.3^ab^	1.3 ± 0.3^abc^	4.8 ± 0.7^c^	3.4 ± 0.5^abc^
L4	4.7 ± 0.4^c^	3.3 ± 0.2^bc^	7.3 ± 0.5^ab^	7.1 ± 0.7^ab^
Hb1	2.8 ± 0.2^ab^	1.9 ± 0.2^ab^	6.2 ± 0.5^abc^	5.0 ± 0.4^abc^
Hb2	3.4 ± 0.3^abc^	1.7 ± 0.3^abc^	7.2 ± 0.3^ab^	6.4 ± 0.7^abc^
Hb3	4.1 ± 0.5^abc^	1.5 ± 0.3^a^	6.1 ± 0.4^abc^	5.8 ± 0.5^abc^
Hb4	3.2 ± 0.4^abc^	1.3 ± 0.4^abc^	7.1 ± 1.0^abc^	6.8 ± 0.5^abc^

*Note:* Genotype codes (H, L, Hb) are described in Table 1. Different letters indicate significant differences (Kruskal–Wallis test with Dunn's post hoc, Bonferroni‐adjusted, *α* = 0.05).

*Abbreviations:* H = *A. glutinosa* with high points of spring phenology (early spring phenology); Hb = hybrids of *A. glutinosa × A. incana*; L = *A. glutinosa* with low points of spring phenology (late phenology).

### 3.3 Alnus Explant Influence on Seed Germination and Plant Phytomorphology

**FIGURE 5:**
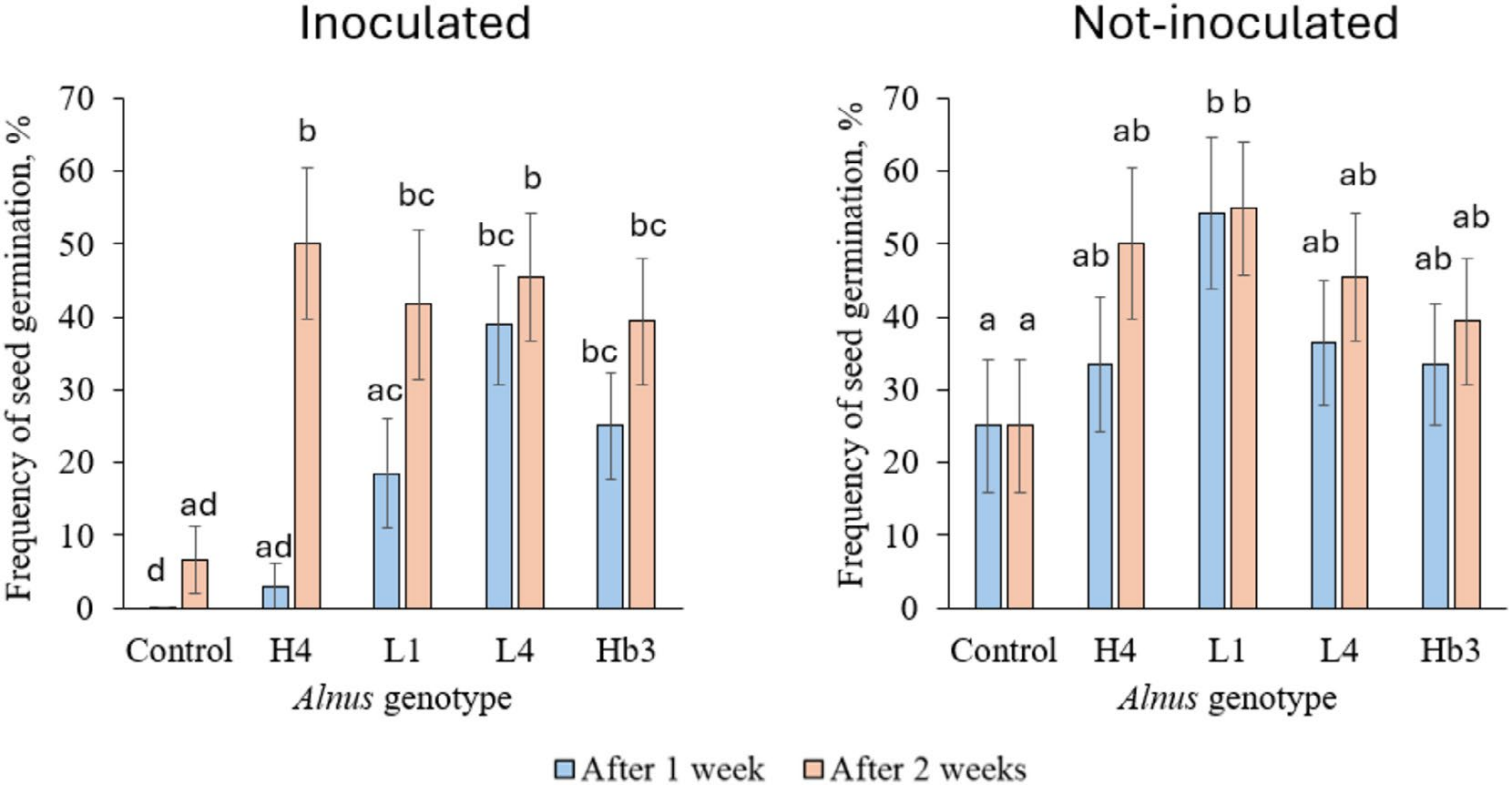
The seed germination 1 and 2 weeks after planting the seeds. On the left—when *Alnus* was growing with inoculated *Cladosporium cladosporioides*; on the right—when *Alnus* was growing without any fungal inoculum. The codes of *Alnus* genotypes are presented in Table 1. H = *Alnus glutinosa* with high spring phenology points (early spring phenology); Hb = hybrids of *A. glutinosa* × *A.* incana; L = *A. glutinosa* with low spring phenology points (late spring phenology). Statistically significant differences (*p* < 0.05) are shown with different letters above error bars representing standard errors (SE). For statistical analysis, Fisher's exact test and the Benjamini–Hochberg (BH) multiple comparisons adjustment were used.

To estimate the ability of different genotypes of *Alnus* explants to induce other *Alnus* seed germination and plant morphological parameters in vitro, four different genotypes of *Alnus* explants, 23–95–5 K (H4), 4–139–4 K (L1), 19–43–8 K (L4) and 026 (Hb3) were selected, having at least one of each broad genotype group. Half the experimental tubes were inoculated with a fungal pathogen, *C. cladosporioides*. The frequency of *Alnus* seed germination was determined 1 and 2 weeks after planting the seeds. All the selected explants of different *Alnus* genotypes increased *Alnus* seed germination frequency in tubes with inoculated *C. cladosporioides* 2 weeks after planting the seeds (by: H4 43.3%, L1 35.0%, L4 38.8% and Hb3 32.7%, compared to the control) (Figure 5). The L4 genotype *Alnus* explants had the largest impact on the induced frequency of seed germination 1 week after planting the seeds (higher by 38.8%, compared to the control). The L1 genotype *Alnus* explants induced the frequency of seed germination 2 weeks after planting the seeds in tubes without the inoculum (by 29.8%) (Figure 5).

*Alnus* phytomorphological parameters (shoot length, the primary root length, the longest lateral root length, and the number of lateral roots) were assessed 8 weeks after planting the seeds. All four selected explants of different *Alnus* genotypes had a significant (*p* < 0.05) positive impact on the primary root length of *Alnus* plants that were inoculated with *C. cladosporioides*: (length increased by 31.3 mm (H4), 53.5 mm (L1), 53.2 mm (L4) and 39.4 mm (Hb3), compared to the control) (Table 3). The L4 genotype *Alnus* explants had the largest positive impact on the lateral root length of *Alnus* plants (length increased by 21.0 mm, compared to the control) inoculated with *C. cladosporioides*. Furthermore, after the inoculation, the L4 genotype *Alnus* explants had the largest positive impact on the number of *Alnus* lateral roots (2.5 times higher lateral root number compared to the control) among all the selected genotypes.

**TABLE 3:** Plant morphological parameters (shoot length, primary root length, lateral root length and number of lateral roots) of *Alnus* plants that were grown together with selected (H4, L1, L4 and Hb3) *Alnus* explants in vitro culture after 8 weeks.

*Alnus* genotype	Shoot length, mm		Primary root length, mm		Lateral root length, mm		Number of lateral roots	
	Not‐inoculated	Inoculated	Not‐inoculated	Inoculated	Not‐inoculated	Inoculated	Not‐inoculated	Inoculated
Control	16.4 ± 3.7^ab^	13.5 ± 1.5^a^	52.4 ± 8.5^bc^	16.5 ± 3.5^a^	42.1 ± 6.5^bc^	13.2 ± 2.5^a^	9.6 ± 3.2^ab^	3.5 ± 0.5^d^
H4	24.0 ± 2.1^bc^	21.3 ± 3.8^abc^	43.1 ± 3.6^b^	47.8 ± 3.0^b^	40.8 ± 2.8^bc^	19.6 ± 3.5^a^	20.8 ± 3.2^c^	5.8 ± 0.5^d^
L1	23.1 ± 0.7^abc^	18.6 ± 1.9^abc^	45.7 ± 3.1^b^	70.0 ± 5.6^c^	43.4 ± 3.4^bc^	18.5 ± 5.4^a^	11.5 ± 2.3^b^	2.6 ± 1.0^d^
L4	27.2 ± 3.4^c^	15.2 ± 1.6^a^	46.1 ± 5.2^b^	60.7 ± 8.5^bc^	53.3 ± 3.9^b^	34.2 ± 3.3^cd^	10.3 ± 1.7^ab^	8.8 ± 1.1^ab^
Hb3	21.6 ± 2.1^abc^	16.3 ± 1.9^ab^	60.1 ± 3.3^bc^	55.9 ± 4.1^bc^	57.0 ± 2.6^b^	22.7 ± 3.9^ad^	12.5 ± 2.5^b^	8.1 ± 1.0^ab^

*Note:* The control was *Alnus* seeds without the *Alnus* explant. The codes of *Alnus* genotypes are presented in Table 1. Different letters indicate significant differences (two‐way ANOVA with Tukey's post hoc test; *α* = 0.05).

Abbreviations: H = *A. glutinosa* with high points of spring phenology (early spring phenology); Hb = hybrids of *A. glutinosa* × *A. incana* ; Inoculated = *Alnus* seeds growing with inoculum of *C. cladosporioides*; L = *A. glutinosa* with low points of spring phenology (late spring phenology); Not‐inoculated = *Alnus* seeds growing without fungal inoculum.

### 3.4 PCA

**FIGURE 6:**
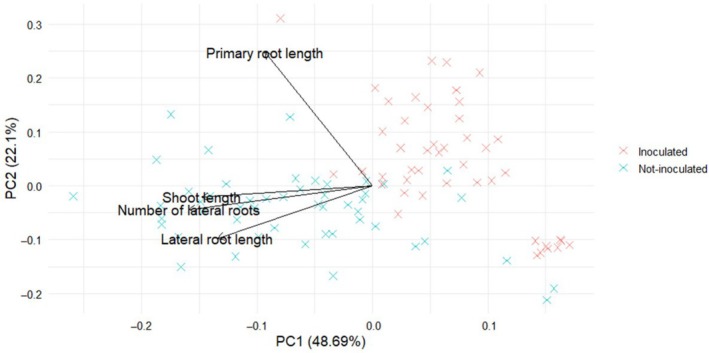
PCA displaying the first (PC1) and second (PC2) components of *Alnus* phytomorphological parameters across all samples. Each point represents an individual replicate, with X‐shaped markers coloured by fungal inoculation status (*Cladosporium cladosporioides*‐inoculated vs. non‐inoculated). Arrows (loading vectors) indicate the contribution and direction of each measured trait: shoot length, primary root length, lateral root length and number of lateral roots. Black vectors and labels represent the correlation strength and direction between traits, illustrating the multivariate relationships among parameters.

PCA was conducted to explore the relationships among four plant growth parameters: shoot length, primary root length, lateral root length and number of lateral roots in both inoculated with *C. cladosporioides* and non‐inoculated samples (Figure 6). The first two principal components (PC1 and PC2) explained 48.7% and 22.1% of the total variance, respectively. The biplot revealed a clear clustering trend where inoculated and non‐inoculated samples occupied partially distinct regions in the PCA space, suggesting that fungal inoculation influenced the multivariate trait profile. Vector directions in the biplot indicated that shoot length, number of lateral roots and lateral root length were positively correlated, with moderate contributions to PC1. Root length, in contrast, contributed more strongly to PC2 and showed a partially orthogonal relationship with the other parameters, indicating a distinct pattern of variation (Figure 6).

## 4 Discussion

Previous studies on *Alnus* (family *Betulaceae*) species *A. glutinosa* and *A. incana* have mainly focused on their distribution range and possible shifts in the future (Douda et al. 2014). Nevertheless, *A. glutinosa* is more common on wet sites, while *A. incana* is more common on drier sites; the warming climate alters their natural habitats, leading to more frequent hybridization (Jurkšienė et al. 2021). Young *Alnus* seedlings are highly susceptible to fungal diseases (Štochlová et al. 2012). The hybrids of *A. glutinosa* × *A. incana* could be more adaptive tree species for better resistance to various pests and pathogens (Štochlová et al. 2016). However, the resistance of *Alnus* trees to biotic and abiotic factors highly depends on their phenotypic plasticity (Pliūra 2004). Thus, testing of potential *Alnus* hybrid genotypes that would characterise better growth and resistance properties is needed. In our study, we analysed four genotypes of *Alnus* hybrid, assessing their disinfection efficiency and *Alnus* hybrid influence to induce seed germination rate and growth parameters of other *Alnus* explants using in vitro screening assays. In comparison, five genotypes of *A. glutinosa* were selected with early spring phenology and four genotypes with late spring phenology.

Although the micropropagation technique allows us to test new tree genotypes, it faces limits and challenges, such as plant contamination with pathogenic fungi that cause damping‐off symptoms (Ďurkovič et al. 2017). In our study, pilot experiments were conducted on the disinfection method and timing of *Alnus* explants to minimise the risk of contamination while minimising the impact on tree development. Comparing *Alnus* based on its early and late spring phenology, as well as *A. glutinosa* to hybrids, no differences were found in disinfection success. However, the success of tree explants in disinfection using tissue culture is primarily determined by tree genotype. Our results revealed that three out of four genotypes of *Alnus* hybrid explants were the least contaminated with pathogenic fungi among all 13 selected *Alnus* genotypes. However, the fourth *Alnus* hybrid genotype had the second‐highest contamination rate. This finding aligns with other studies demonstrating that tree hybrids can also be less resistant to contamination when assessed using in vitro screening assays, depending on their gene pools (Hoenicka and Fladung 2006). Tree hybrids may carry more endophytic fungi from both parental species, potentially raising the risk of contamination in micropropagation (Jaiswal et al. 2023).

The susceptibility of plants to fungal pathogens can change as they progress through different phenological phases (Hurel et al. 2021). Plants characterised by quicker growth and development could also be more susceptible to pathogens since their growth‐defence trade‐offs can explain their use of inner resources for growth versus defence and resistance (He et al. 2022). These mechanisms have been determined in greenhouse conditions (Čėsnienė et al. 2024), and deep in vitro analysis, including protein signals, has been performed (Lee 2024). Our results align with these studies, showing that without contamination of pathogenic fungus, the explants of *Alnus* hybrids 047 (Hb2) and 026 (Hb3) were characterised by higher leaf emergence than the explants of *A. glutinosa* genotype 19–43–8 K (L4). However, the highest tolerance to pathogenic fungi was determined in *A. glutinosa* genotype 19–43–8 K (L4). These L4 explants exhibited higher leaf emergence, a greater number of leaves per explant, and increased leaf width. It confirms that hybrid plants might grow and develop faster than *A. glutinosa* but become more susceptible to fungal contamination and less tolerant to pathogens. However, it depends on the specific tree genotype. Analysis of *Alnus* spring phenology revealed that genotypes with early spring phenology exhibited a higher rate of leaf emergence in explants compared to those with late spring phenology. This may reflect inherent differences in developmental timing and metabolic activity, as early‐flushing genotypes often possess a more rapid reactivation of meristematic growth and hormonal signalling in response to culture conditions (Le Provost et al. 2023), which can enhance regeneration and growth performance in vitro.

Another important factor to consider when selecting tree genotypes is their influence to induce the growth of other trees by cooperating through a fungal web or in a specific manner (Simard 2018). To assess *Alnus* explant properties to induce other *Alnus* seed germination and to improve plant morphological parameters, we selected four *Alnus* genotypes that showed the best performance in the contamination experiment and also differed by spring phenology points: one with high phenology points (23–95–5 K [H4]), two with low spring phenology points (4–139–4 K [L1] and 19–43–8 K [L4]), and one *Alnus* hybrid (026 [Hb3]). In the previous study, it was found that the seed germination success of *Alnus* species may be positively dependent on greater seed mass and larger seed size (Gomes Marques et al. 2022). Our study showed that the seed germination rate also depends on neighbouring plant explants and their genotype. For example, the explant of *Alnus* genotype 4–139–4 K (L1) increased the seed germination rate of *A. glutinosa* the most. The explants of other selected *Alnus* genotypes also had a trend to induce *Alnus* seed germination.

Climate warming leads to more severe forest damage, increasing the frequency of droughts and winds, and consequently, attacks by pests and pathogens (Jactel et al. 2019). One of the largest threats to *Alnus* seed germination is the increased abundance of pathogenic fungi (Haque 2014). In this context, *Alnus* properties to induce other trees' growth based on *Alnus* genotype should be considered before afforestations and reforestations (Zhang et al. 2022). We found that the explants of all selected *Alnus* genotypes increased neighbouring *Alnus* seed germination when the tubes were inoculated with an opportunistic pathogen *C. cladosporioides*. Furthermore, the explants of all four selected *Alnus* genotypes increased the primary root length of neighbouring *Alnus* plants. *C. cladosporioides* is widely recognised as a fungal species with a broad ecological range, often existing as an endophyte in various plant hosts, including mature *Alnus* trees, where it typically remains asymptomatic and non‐threatening (Kowalski and Kehr 1992). However, our findings support previous research suggesting that under specific conditions—such as in young, stressed or immunologically immature seedlings—*C. cladosporioides* may shift from being an endophyte to an opportunistic pathogen (Dutta et al. 2014). This behaviour is particularly concerning in early developmental stages, such as in seed orchards, where the fungus can impair root development and hinder nitrogen exchange between the plant and rhizosphere (Kranjec Orlović et al. 2020; Põlme et al. 2013). It was also determined that under the stress from *C. cladosporioides*, the explants of *A. glutinosa* genotype 19–43–8 K (L4) and *Alnus* hybrid genotype 026 (Hb3) had the largest effect on increased lateral root length and number of lateral roots of neighbouring *Alnus* plants.

PCA revealed that inoculation with *C. cladosporioides* induced coordinated shifts in several key growth traits of young *Alnus* seedlings, supporting the notion that fungal presence can significantly reshape early developmental dynamics. Traits such as shoot length, lateral root length, and number of lateral roots clustered together along PC1, suggesting a shared response pathway or developmental coupling under fungal stress. In contrast, primary root length loaded distinctly on PC2, indicating a more independent or variable response. This divergence aligns with previous findings that root system architecture, particularly the balance between primary and lateral root growth, can be highly plastic under biotic stress (Muthert et al. 2020). The observed compensatory growth in lateral roots and shoots may represent an adaptive or stress‐induced response aimed at maintaining nutrient uptake and establishment in the presence of mild pathogenic pressure (Devi et al. 2017). These findings highlight the context‐dependent behaviour of fungal endophytes (Mengistu 2020) and emphasise the vulnerability of early developmental stages to shifts in microbial interactions. However, while in vitro screening assays offer a controlled and replicable environment to study plant–microbe interactions, they may not fully capture the complexity of natural conditions. Nonetheless, these assays provide a valuable first step in identifying promising *Alnus* genotypes with desirable traits, which can be prioritised for follow‐up field trials. Such studies are essential to validate the ecological relevance of the in vitro findings and to assess how genotype‐dependent responses translate to plant performance and pathogen susceptibility in real‐world environments.

## 5 Conclusions

*A. glutinosa* genotype 19–43–8 K had the best disinfection efficiency and was the most tolerant to an opportunistic pathogen using in vitro screening assays. All selected genotypes of *Alnus* explants increased seed germination of neighbouring *Alnus* and induced plant development under stress caused by *C. cladosporioides*. The *Alnus* explants of genotypes 19–43–8 K (late spring phenology) and *Alnus* hybrid 026 influenced an increase in neighbouring *Alnus* lateral root length and the number of lateral roots the most. Further studies on *Alnus* secondary metabolites and the interaction between *Frankia* spp. bacteria and tree tolerance to pathogenic fungi should be performed with these selected *Alnus* genotypes.

## Author Contributions

**Vytautas Čėsna:** conceptualisation, investigation, writing – original draft, methodology, validation, visualisation, software, formal analysis, data curation. **Ieva Čėsnienė:** writing – review and editing, conceptualisation, formal analysis, visualisation. **Virgilijus Baliuckas:** writing – review and editing, data curation, resources, formal analysis. **Jonas Žiauka:** writing – review and editing, methodology, conceptualisation, validation, formal analysis, data curation.

## Conflicts of Interest

The authors declare no conflicts of interest.

## Supporting information


**Data S1:** Supporting Information.

## Data Availability

The data that supports the findings of this study are available in the Supporting Information of this article.
