# Supplementary material for: Disinfection Efficiency Among Black Alder and Hybrid Alder Genotypes and Their Influence on Alder Seed Germination In Vitro
Source: Environ Microbiol Rep. 2025 Sep 19;17(5):e70183. doi: 10.1111/1758-2229.70183 (PMC12447240; doi:10.1111/1758-2229.70183)
Supplement: Supplementary file 1 — Data S1: Supporting Information. [file EMI4-17-e70183-s001.pdf]

Supplementary Table 1. The results of pilot experiments evaluating the effects of different pH levels (3.7, 4.3, 4.9, and 5.5) in WPM medium on *Alnus* seed germination and contamination rates. Each condition was assessed for germination percentage and contamination 10 days after planting

pH 3.7	Seed germination, %	20.0±5,25
	Contamination, %	60.0±10,25
pH 4.3	Seed germination, %	25.0±4.30
	Contamination, %	60.0±10,25
**pH 4.9**	**Seed germination, %**	**50.00±8.33**
	**Contamination, %**	**20.0±2.25**
pH 5.5	Seed germination, %	25.0±4.30
	Contamination, %	60.0±10,25

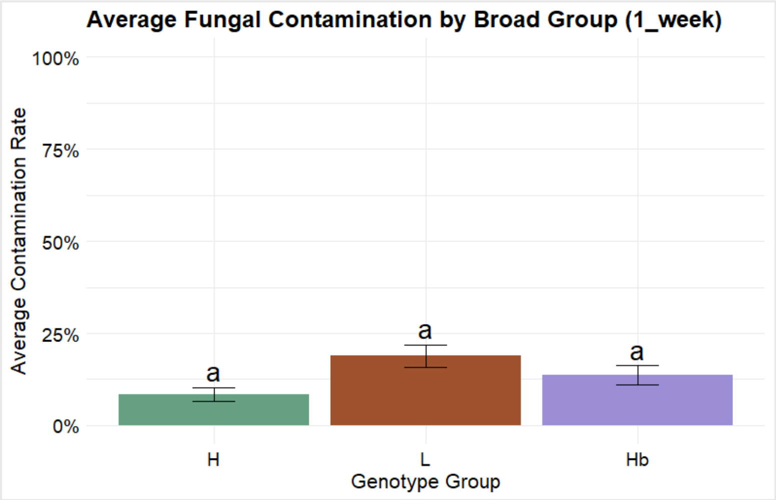

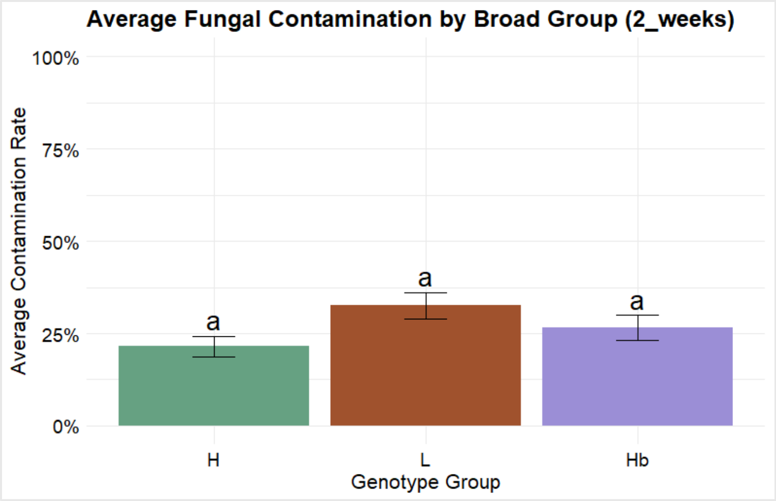

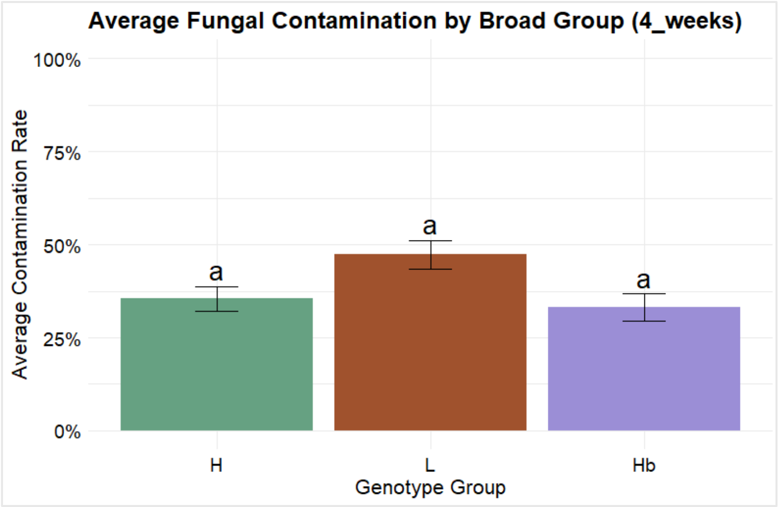

Supplementary Figure 1. Frequency of fungal contamination (%) in different broad genotype groups (different colors) of vegetative *Alnus* explants: (H) *A. glutinosa* with high points of spring phenology (early spring phenology); (L) *A. glutinosa* with low points of spring phenology (late phenology); (Hb) hybrids of *A. glutinosa* × *A. incana* at two, and four weeks after planting into WPM medium. Statistically significant differences ($p < 0.05$) are shown with different letters above error bars representing standard errors (SE). For statistical analysis, Fisher's exact test and the Benjamini-Hochberg (BH) multiple comparisons adjustment were used

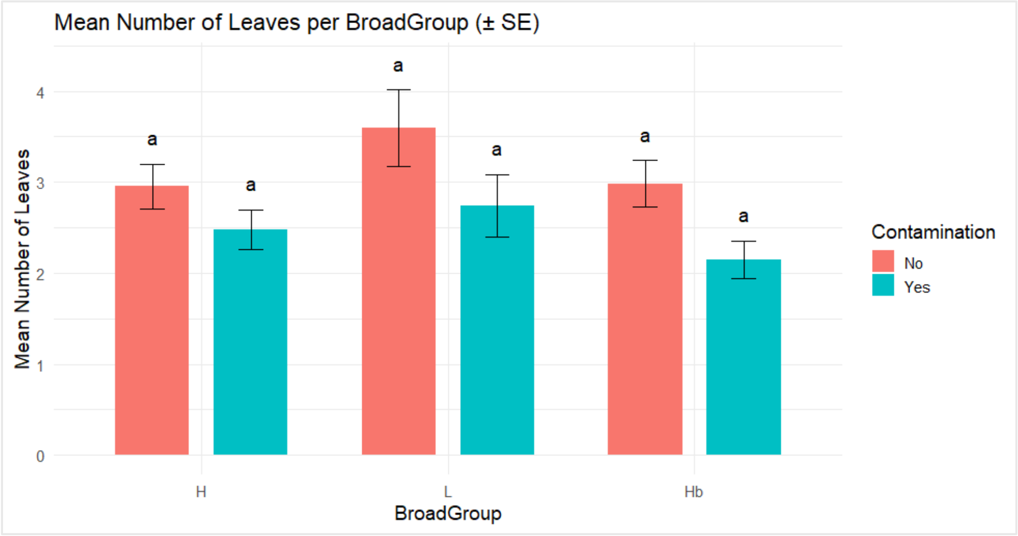

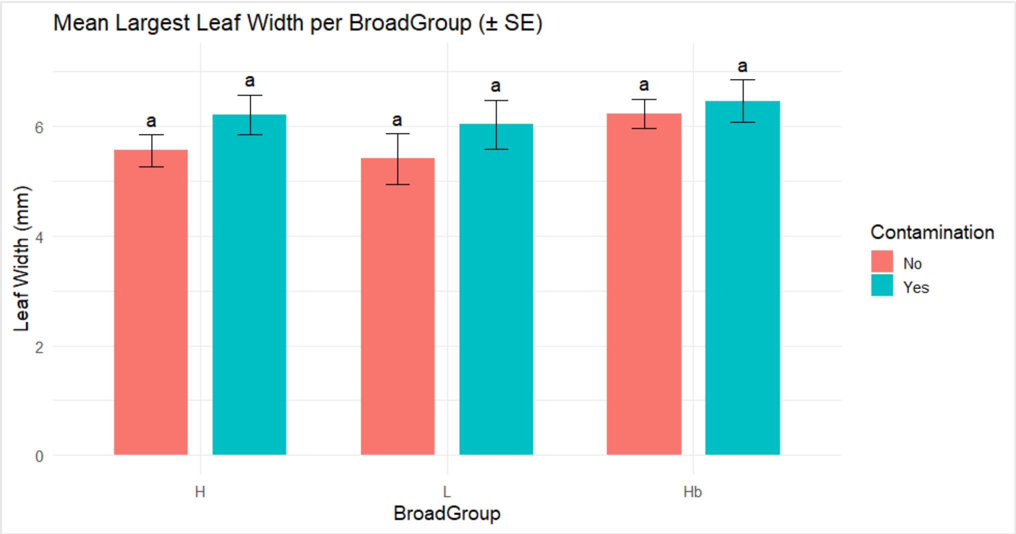

Supplementary Figure 2. Mean number of leaves and the largest leaf width in different broad genotype groups of vegetative *Alnus* explants: (H) *A. glutinosa* with high points of spring phenology (early spring phenology); (L) *A. glutinosa* with low points of spring phenology (late phenology); (Hb) hybrids of *A. glutinosa* × *A. incana* at eight weeks after planting into WPM medium. Statistically significant differences ($p < 0.05$) are shown with different letters above error bars representing standard errors (SE) (Kruskal–Wallis test with Dunn's post hoc, Bonferroni-adjusted, $\alpha = 0.05$)
